# Drug-related problems in community-dwelling primary care patients screened positive for dementia

**DOI:** 10.1017/S1041610217001442

**Published:** 2017-08-07

**Authors:** D. Wucherer, J. R. Thyrian, T. Eichler, J. Hertel, I. Kilimann, S. Richter, B. Michalowsky, I. Zwingmann, A. Dreier-Wolfgramm, C. A. Ritter, S. Teipel, W. Hoffmann

**Affiliations:** 1 German Center for Neurodegenerative Diseases (DZNE), Rostock/Greifswald, Greifswald, Germany; 2 Department of Psychiatry and Psychotherapy, University Medicine Greifswald, Greifswald, Germany; 3 German Center for Neurodegenerative Diseases (DZNE), Rostock/Greifswald, Rostock, Germany; 4 Department of Psychosomatic Medicine, Rostock University Medical Center, Rostock, Germany; 5 Institute for Community Medicine, Section Epidemiology of Health Care and Community Health, University Medicine Greifswald, Greifswald, Germany; 6 Institute of Pharmacy, Section Clinical Pharmacy, University of Greifswald, Greifswald, Germany

**Keywords:** primary care, dementia, DelpHi-MV study, drug-related problem, medication review

## Abstract

**Background:**

Older people have a higher risk of drug-related problems (DRPs). However, little is known about the prevalence of DRPs in community-dwelling people who screened positive for dementia. Our study aimed to determine (1) the prevalence and types of DRPs and (2) the socio-demographic and clinical variables associated with DRPs in people screened positive for dementia in primary care.

**Methods:**

The Dementia: life- and person-centered help in Mecklenburg-Western Pomerania (DelpHi-MV) study is a general practitioner (GP)-based cluster-randomized controlled intervention study to implement and evaluate an innovative concept of collaborative dementia care management in the primary care setting in Germany. Medication reviews of 446 study participants were conducted by pharmacists based on a comprehensive baseline assessment that included a computer-based home medication assessment. ClinicalTrials.gov Identifier: NCT01401582.

**Results:**

A total of 1,077 DRPs were documented. In 414 study participants (93%), at least one DRP was detected by a pharmacist. The most frequent DRPs were administration and compliance problems (60%), drug interactions (17%), and problems with inappropriate drug choice (15%). The number of DRPs was significantly associated with the total number of drugs taken and with a formal diagnosis of a mental or behavioral disorder.

**Conclusions:**

Degree of cognitive impairment (MMSE defined) and formal diagnosis of dementia were not risk factors for an increased number of DRPs. However, the total number of drug taken and the presence of a diagnosis of mental and behavioral disorders were associated with an increased total number of DRPs.

## Introduction

Approximately 75% of the 1.5 million people with dementia (PwD) in Germany are community-dwelling patients (Grass-Kapanke *et al.*, 2008). Most of them (up to 77%) are affected by multiple chronic diseases and are treated with complex pharmacotherapy regimes (up to seven chronic-use drugs per patient) that are associated with drug-related problems (DRPs) (Elliott *et al.*, 2015; Gustafsson *et al.*, 2016; Wucherer *et al.*, 2016). The Pharmaceutical Care Network Europe defines a DRP as “an event or circumstance involving drug therapy that actually or potentially interferes with desired health outcomes” (Pharmaceutical Care Network Europe Foundation, 2010). DRPs include drug–drug interactions, an over- or under-supply of medication, non-compliance, application errors, inadequate self-medication, adverse drug reactions, and drug abuse. DRPs can lead to increase in morbidity, reduction in quality of life, medication-related hospital admissions, and higher healthcare costs (Leendertse *et al.*, 2011; Gustafsson *et al.*, 2016). Advanced age and impaired cognition increase the risk of DRPs; a prospective multicenter study from the Netherlands identified impaired cognition as one of the main determinants of preventable medication-related hospital admissions in the general population (Leendertse *et al.*, 2008). To date, little is known about the association between dementia diseases and the presence of DRPs in primary care. A high prevalence of DRPs in this population would drive the inclusion of systematic medication reviews in dementia care programs. Identifying risk factors for DRPs would help allocate medication review resources to the population at the highest risk. Accordingly, the goals of the present analysis were to determine (1) the frequency and the type of DRPs and (2) the socio-demographic and clinical variables associated with DRPs in people who screened positive for dementia in a German community-dwelling setting.

## Methods

### Study design and data collection

The present cross-sectional analysis was based on data from the Dementia: life- and person-centered help in Mecklenburg-Western Pomerania (DelpHi-MV) study, a GP-based, cluster-randomized, controlled intervention study to implement and evaluate an innovative concept of collaborative dementia care management in Germany (ClinicalTrials.gov Identifier: NCT01401582). More details about the DelpHi-MV study was published elsewhere (Thyrian et al., 2012; Thyrian et al., 2016). The age of the patients was 70 years or older; they lived at home, screened positively for dementia with DemTect (<9) (Calabrese and Kessler, 2000) as an inclusion criterion in participating GP practices, and provided a written informed consent for participation in the study. If a patient was unable to give written informed consent, the form was signed on his or her behalf by his or her legal representative (as approved by the Ethical Committee of the Chamber of Physicians of Mecklenburg-Western Pomerania, registry number BB 20/11). A comprehensive standardized baseline assessment was conducted at the participant's home by study nurses with dementia-specific qualifications and included a computer-assisted home medication assessment. Detailed description of home medication assessment in DelpHi-MV study was published by Fiss et al. (2013). The study nurses (*n* = 6) were trained by the study pharmacists (*n* = 2) to perform the medication assessment. The structured training included information about the principles of drug administration, pharmacotherapy for older patients, and DRPs. For the computer-assisted home medication assessment, the study nurses also judged medication storage, timeliness of the medication list, necessity of the pill dispenser, and the abilities of the study participants or caregivers to manage the medication by themselves.

### Participants

A total of 6,838 patients was screened for dementia in 125 GP practices. Of these, 1,166 patients (17%) were eligible for the DelpHi-MV study, 634 patients (54%) agreed to participate. One hundred and eighteen patients dropped out of the study before the baseline assessment due to withdrawal of informed consent: *n* = 85, death: *n* = 19, relocation: *n* = 5, or other reasons: *n* = 9, and 516 participants started the baseline assessment. A total of 70 participants was excluded from the present analyses during the period of baseline assessment due to the missing data (missing data: *n* = 46; death: *n* = 2; withdrawal of the informed consent: *n* = 18; moving away: *n* = 1; not assessed: *n* = 1; other reasons: *n* = 2). Accordingly, the present analysis was based on the data of 446 participants of the DelpHi-MV study with a complete baseline medication review.

There were no significant differences in age, sex, or the DemTect score among patients included in the analysis (*n* = 516) and those who dropped out of the study before baseline assessment (*n* = 118) (see Table S1 available as supplementary material online attached to the electronic version of this paper at http://journals.cambridge.org/ipg). Furthermore, no significant differences were observed in age, sex, or DRPs between the analyzed patients and those who were excluded because of missing data in any covariate included in the analyses. However, patients excluded from the analyses due to missing data showed lower DemTect scores compared to patients included in the analyses (DemTect score 6.1 (SD = 1.90) vs. 4.5 (SD = 2.08), *p* = 0.001) (see Table S2 available as supplementary material online attached to the electronic version of this paper at http://journals.cambridge.org/ipg).

### Data analyses

A total of 371 (83.2%) medication reviews for our analysis was conducted by the study pharmacists (*n* = 2), and 75 (16.8%) medication reviews were conducted by pharmacists from the study's participating public pharmacies. All pharmacists prepared the medication review independently and identified existing and potential DRPs. The study pharmacists (*n* = 2) trained the pharmacists (*n* = 37) in the study's participating public pharmacies (*n* = 29). The structured training included the following aspects: pharmacotherapy for older patients, special cases of pharmacotherapy in dementia diseases, DRPs, DelpHi-MV study structure, implementation of medication review, and special features of documentation. The training materials were given to the pharmacists in the form of a portfolio; the pharmacists also had the ability to consult the study pharmacist by phone.

The home medication assessment examined the study participant's entire medication history (prescription drugs and over-the-counter (OTC) drugs) including compliance, adverse effects, and drug administration (Fiss *et al.*, 2013). A community pharmacist or the pharmacist in the study center conducted the medication review for the study's participants. Active substances were coded according to the Anatomical Therapeutic Chemical (ATC) classification system (WIdO (Wissenschaftliches Institut der AOK), 2016). Topical agents and homeopathic medicines were not considered in this analysis. The DRPs were grouped into five main groups according to the PIE-Doc®-System (Schaefer, 2002): inappropriate drug choice; inappropriate administration by patients/problems with administration and compliance; inappropriate dosage/problems with the dosage; problems with drug interactions; and problems with adverse drug events (ADEs). Drug interactions, drug–food interactions, and double prescriptions (of the same drugs or of the drugs in the same drug class) were identified by the Risk-Check tool CAVE of the ABDA-Database. A “traffic light system,” a pragmatic system of DDI classification of the ABDA-Database, was employed to classify the drug interactions and drug–food interactions into six categories of severity: “serious consequences probable, contraindicated,” “contraindicated as a precaution,” “monitoring or adjustment is needed,” “monitoring and adjustment is necessary in some cases,” “supervise as a precaution,” and “no action is normally required” (Pharma-Daten-Service, 2017). The first three categories of severity were considered during medication reviews. The clinical relevance of drug–drug interactions was assessed by the pharmacists during the medication review implementation. In the analysis, the interactions of category “monitoring or adjustment is needed” were described as “potential drug interactions of moderate severity.” The interactions of categories “serious consequences probable, contraindicated” and “contraindicated as a precaution” were summed as “potential drug interactions, clinically relevant.” Potentially inappropriate medications (PIMs), the drugs for which the risk of an ADE outweighs the clinical benefit, particularly when there is an evidence in favor of a safer or more effective alternative therapy for the same condition (Laroche *et al.*, 2009), were determined using a list of PIM in the elderly (Priscus list). The German Priscus list was established in line with the international PIM lists and published in 2010, aiming to reduce the rate of ADE and to provide higher medication safety (Holt *et al.*, 2010).

To analyze the associations between DRPs and socio-demographic and clinical variables, the following variables were considered: age, sex, support with medication (yes/no), cognitive status, functional status, depressive symptoms, visit to a specialist (neurologist/psychiatrist (yes/no)), total number of drugs taken, formal diagnosis of dementia, diagnosis of mental and behavioral disorders, and number of comorbid diagnoses. The severity of cognitive impairment was evaluated using the Mini-Mental State Examination (MMSE) (Kessler *et al.*, 1990). The following categories for the severity were applied: “no indication of cognitive impairment” (score 27–30) and “mild” (20–26), “moderate” (10–19), or “severe” (0–9) cognitive impairment (Deutsche Gesellschaft für Psychiatrie und Psychotherapie, Psychosomatik und Nervenheilkunde (DGPPN), 2016). The Geriatric Depression Scale (GDS) was used to assess depressive symptoms which were categorized as dichotomized variable in two categories “no depression” (score 0–5) and “possible depression” (score 6–15) (Gauggel and Birkner, 1999). The functional status was assessed using the Bayer Activities of Daily Living Scale (B-ADL) (Hindmarch *et al.*, 1998) with a mean score between 1 and 10, where 1 indicates the lowest and 10 indicates the highest possible impairment. According to the International Classification of Diseases and Related Health Problems (ICD-10, German Modification) (Deutsches Institut für medizinische Dokumentation und Information (DIMDI), 2011), medical diagnoses were retrieved from the participants’ medical records of an individual patient's GP. A dementia diagnosis refers to any of the following ICD-10 codes: F00/G30 (dementia due to Alzheimer's disease), F01 (vascular dementia), F02 (dementia in other diseases), F03 (unspecified dementia), or G31 (other degenerative diseases of nervous system, not otherwise classified). Diagnosis of mental and behavioral disorders refers to the ICD-10 codes F04-F69.

### Statistical analyses

We fitted Poisson regression models to evaluate which variables were associated with the total number of DRPs. The regression model included the degree of cognitive impairment (MMSE defined) as an explanatory variable. Age, sex, living situation (dichotomous: living alone or not alone), functional status (measured with Bayer-ADL), depressive symptoms (measured with GDS), total number of drug taken, documented diagnosis of dementia before screening (dichotomous: having a dementia diagnosis or not), the number of somatic comorbidities (as total number from the medical records), and diagnosis of mental and behavioral disorders (dichotomous: having a diagnosis or not) were included as covariates. Whereas socio-demographic factors and the total number of drugs taken were included to attenuate possible confounding factors, the clinical variables were the predictors of interest. To account for the clustering of participants who were recruited by the same GP, we included random effects of the GP in the Poisson regression model. For sensitivity analyses, we ran a mixed effect negative binomial regression with the same specifications. Before running the final regression model, we checked for non-linear relations using the multivariate fractional polynomial procedure (Royston and Sauerbrei, 2008). However, we found no indication of non-linear relationships. Furthermore, by using exploratory analyses, we checked the associations of the same predictors with problems (dichotomous: prevalent vs. not prevalent) in single categories with analogous logistic regressions. All regression analyses were performed in the remaining 446 cases belonging to 90 clusters (unequal sample sizes per cluster). The standard errors of the regression coefficients were estimated using the jackknife technique, which provides appropriate estimates of standard errors in complex samples (Efron and Tibshirani, 1986). Statistical analyses were performed using STATA®13 (StataCorp, 2014).

## Results

### Socio-demographic and clinical characteristics of the study sample

The socio-demographic and clinical characteristics of the study sample for this analysis are represented in Table 1. More detailed characteristics of the whole study sample have been published by Thyrian *et al.* (2016).

**Table 1.** Socio-demographic and clinical characteristics of study sample

	community-dwelling people screened					
	positive for dementia:					
	total	without DRP	with DRP	*t*	df	*p*
	*n* = 446	*n* = 31	*n* = 415			
Age, mean (SD)	79.8(5.4)	81.2(6.9)	79.7(5.3)	1.184	32.906	0.244^a^
Sex (female), *n* (%)	257(57.6)	17(54.8)	240(57.8)			0.851^b^
Degree of cognitive impairment (MMSE defined), mean (SD)	22.5(5.0)	21.0(4.7)	22.7(5.0)	−1.848	35.686	0.073^a^
No cognitive impairment (score, 27–30)	106(23.8)	4(12.9)	102(24.6)			
Mild cognitive impairment (score, 20–26)	235(52.7)	16(51.6)	219(52.8)			
Moderate cognitive impairment (score, 10–19)	96(21.5)	11(35.5)	85(20.5)			
Severe cognitive impairment (score, 0–9)	9(2.0)	0(0.0)	9(2.2)			0.184^b*^
Formal diagnosis of dementia (ICD-10: F00-F03/G30/G31) (yes), *n* (%)	166(37.2)	13(41.9)	153(36.9)			0.569^b^
Diagnosis of mental and behavioral disorders (ICD-10: F04-F69) (yes), *n* (%)	116(25.9)	6(19.3)	110(26.5)			0.628^b^
Previous visit to specialist (neurologist/psychiatrist), *n* (%)	115(25.8)	5(16.1)	110(26.5)			0.287^b^
Support with medication (yes), *n* (%)	93(20.9)	6(19.4)	87(21.0)			1.000^b^
Comorbid diagnoses, mean (SD)	12.1(7.3)	10.8(5.7)	12.2(7.4)	−1.310	38.519	0.198^a^
Total no. of drugs, mean (SD)	6.4(3.2)	4.4(2.9)	6.6(3.2)	−4.015	35.860	**0.001^a^**
Living alone (yes), *n* (%)	220(49.3)	14(45.2)	206(49.6)			0.711^b^
Depression (GDS) (yes), *n* (%)	72(16.1)	3(9.7)	69(16.6)			0.448^b^
Functional status (B-ADL), mean (SD)	3.6(2.4)	4.0(2.7)	3.6(2.4)	0.905	33.932	0.371^a^

Standard deviations or percentages are in brackets. MMSE, Mini-Mental State Examination (range 0–30; higher score indicates better cognitive functioning); B-ADL, Bayer Activities of Daily Living Scale (range 0–10; lower score indicates better performance); GDS, Geriatric Depression Scale (sum score 0–15; score ≥6 indicates depression); ^a^Welch's *t*-test, ^b^Fisher's exact test; *Fisher's exact test calculation for all MMSE-categories; bold *p*-value indicates *p* < 0.05; df, degrees of freedom; *t*, *t*-statistic of the Welch's *t*-test.

### Drug-related problems

**Figure 1.**
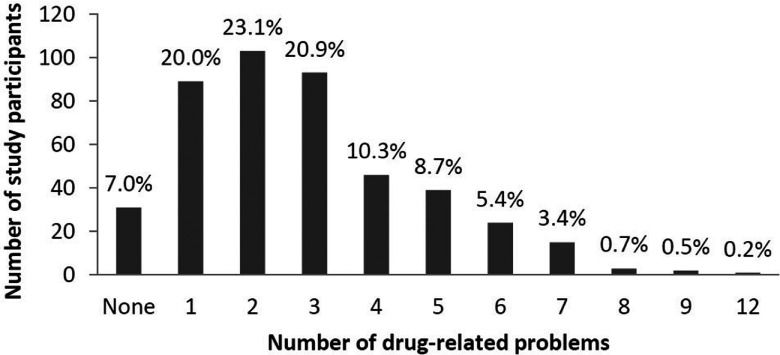
Prevalence of drug-related problems in the study samples (percentages may not sum to 100, because of rounding).

Polypharmacy, defined here as the use of five or more prescription medications that was to be taken according to a fixed schedule (none “*pro re nata*” medication), was identified in 67.3% (*n* = 300) of the study participants. Of the 446 total patients, a 414 (92.8%) had at least one DRP (Figure 1) detected by a pharmacist (in the community pharmacies or in the study center) or a study nurse during a home visit. Almost two-thirds of the study participants (*n* = 286/446; 64.1%) had one to three detected DRPs, and almost one-third of the study participants (*n* = 122/446; 27.3%) had four to seven DRPs. Six study participants (1.4%) had eight to twelve DRPs. The mean time required for the medication review of each patient was about 25 minutes (SD = 18 minutes).

A total of 1,077 DRPs were registered. Problems related to administration and compliance were the most common group of DRPs (59.9% of registered DRPs; *n* = 645), followed by problems with drug interactions (16.7%; *n* = 180), problems with inappropriate drug choice (14.7%; *n* = 158), problems with the dosage (6.2%; *n* = 67), and problems with ADEs (2.5%; *n* = 27). The most frequent specific DRPs included the following problems: inadequate drug storage (195 of 446 study participants; 43.7% of all study participants in our analysis), inappropriate time of application (*n* = 180/446; 40.4%), inappropriate combination of drugs (*n* = 155/446; 34.8%), no medication list/medication list outdated (*n* = 110/446; 24.7%), inappropriate drugs according to the Priscus list (*n* = 105/446; 22.9%), and forgetting to take the drug (*n* = 82/446; 18.4%) (Table 2). Two percent of the study participants (*n* = 9) took cholinesterase inhibitors and anticholinergic drugs (quetiapine: *n* = 5 cases; amitriptyline: *n* = 3; doxepin, *n* = 1; tolterodine, *n* = 1) at the same time.

**Table 2.** Distribution of drug-related problems, according to the PIE-Doc®-System

		study participants
main group of DRPs	specific DRPs	with DRP ^1^ No ^2^ . (%)
Inappropriate drug choice	Contraindication	3 (0.7)
	Double prescription of active ingredient	16 (3.6)
	Double prescription of therapeutic group	13 (2.9)
	Inappropriate drug form	15 (3.4)
	Inappropriate drug according to Priscus list	102 (22.9)
	Untreated indication	9 (2.0)
Administration and compliance	Inappropriate duration of application: too long	22 (4.9)
	Inappropriate duration of application: too short	2 (0.5)
	Inappropriate time of application	180 (40.4)
	No intake due to forgetfulness	82 (18.4)
	Multiple drug taking	22 (4.9)
	Not drug use by physical complaints	6 (1.4)
	Self-omission of the drug by the patient	23 (5.2)
	Inadequate storage	184 (41.3)
	Inadequate storage in refrigerator	11 (2.5)
	No medication list/medication list is outdated	110 (24.7)
	Use beyond expiration date	1 (0.2)
	Lack of knowledge on correct application	1 (0.2)
Dosage	Drug dose too high	36 (8.1)
	Inappropriate timing of dosing intervals	10 (2.2)
	Dosage increase with good tolerability recommendable	21 (4. 7)
Drug interactions	Potential drug interactions of moderate severity	155 (34.8)
	Potential drug interactions, clinically relevant	14 (3.1)
	Inappropriate combination of drugs with food	11 (2.5)
Adverse drug events	Adverse drug events	27 (6.1)

1 Duplicate entry was possible.

2 One case of DRP was defined as the occurrence of the problem in a study participant. The case with drug–drug interactions means, for example, that a study participant has the drug–drug interactions, regardless of the number of interactions.

The comparison the rate and classification of DRPs detected by the study pharmacists versus the trained public pharmacists showed only one significant difference, the public pharmacists registered more DDIs. For DDI detection in both cases, one and the same database (ABDA) was used, the public pharmacists have recorded more DDI with lower severity.

### Factors associated with drug-related problems

The results of the multivariate logistic regression analyses (*n* = 446 study participants assigned to *n* = 90 clusters) are shown in Tables 3 and 4. The results of the multivariate logistic regression analysis for different main groups of DRPs (Table 3) revealed that degree of cognitive impairment (MMSE defined) was associated with ADEs reported by the study participants (OR: 1.20; 95% CI: 1.06–1.36; *p* = 0.004). The total number of drugs taken (OR: 1.26; 95% CI: 1.15–1.39; *p* < 0.001) and support with medication (OR: 1.78; 95% CI: 1.05–3.02; *p* = 0.033) were associated with drug interactions. The presence of a diagnosis of mental and behavioral disorders was associated with problems of inappropriate drug choice (OR: 1.66; 95% CI: 1.24–2.21; *p* = 0.001; significant regression model for problems with inappropriate drug choice: *χ*^2^(10) = 33.30, *p* < 0.001; problems with ADEs: *χ*^2^(10) = 19.38, *p* = 0.036; problems with interactions: *χ*^2^(10) = 56.15, *p* < 0.001).

**Table 3.** Factors associated with main groups of DRPs

	main group of DRPs	OR	*p*	95%	CI
Sex (female)	Inappropriate drug choice	1.16	0.527	0.72	1.89
	Administration and compliance	1.21	0.505	0.70	2.09
	Dosage	0.63	0.182	0.32	1.25
	Adverse drug events	0.71	0.459	0.29	1.74
	Interactions	0.85	0.488	0.54	1.35
Age (years)	Inappropriate drug choice	1.00	0.766	0.96	1.05
	Administration and compliance	0.97	0.177	0.81	1.04
	Dosage	0.94	0.071	0.88	1.01
	Adverse drug events	1.07	0.136	0.98	1.16
	Interactions	0.99	0.524	0.94	1.03
Functional status (B-ADL)	Inappropriate drug choice	0.94	0.280	0.84	1.05
	Administration and compliance	0.92	0.177	0.81	1.04
	Dosage	1.07	0.375	0.85	1.25
	Adverse drug events	1.21	**0.049**	1.00	1.47
	Interactions	0.99	0.791	0.88	1.10
Degree of Cognitive Impairment (MMSE defined)	Inappropriate drug choice	1.01	0.776	0.95	1.06
	Administration and compliance	1.02	0.570	0.96	1.08
	Dosage	1.03	0.447	0.95	1.11
	Adverse drug events	1.20	**0.004**	1.06	1.36
	Interactions	1.01	0.795	0.96	1.08
Total number of drugs	Inappropriate drug choice	1.16	**0.001**	1.08	1.26
	Administration and compliance	1.03	0.456	0.95	1.13
	Dosage	1.07	0.216	0.96	1.18
	Adverse drug events	1.13	0.063	0.99	1.29
	Interactions	1.32	**0.001**	1.21	1.43
Depression (GDS) (yes)	Inappropriate drug choice	1.74	0.068	0.96	3.16
	Administration and compliance	1.30	0.484	0.63	2.67
	Dosage	0.82	0.657	0.34	1.98
	Adverse drug events	1.54	0.427	0.53	4.42
	Interactions	1.06	0.840	0.58	1.93
Formal diagnosis of dementia (ICD-10: F00-F03/G30/G31) (yes)	Inappropriate drug choice	0.85	0.506	0.52	1.38
	Administration and compliance	0.87	0.615	0.50	1.50
	Dosage	1.43	0.294	0.73	2.79
	Adverse drug events	0.65	0.382	0.24	1.72
	Interactions	1.29	0.281	0.81	2.07
Support with medication (yes)	Inappropriate drug choice	0.85	0.583	0.48	1.50
	Administration and compliance	0.49	**0.019**	0.27	0.89
	Dosage	1.71	0.158	0.81	3.63
	Adverse drug events	0.42	0.187	0.12	1.52
	Interactions	1.78	**0.033**	1.05	3.02
Total number of comorbid diagnoses	Inappropriate drug choice	0.98	0.283	0.95	1.02
	Administration and compliance	1.01	0.607	0.97	1.06
	Dosage	0.97	0.255	0.93	1.02
	Adverse drug events	0.98	0.546	0.92	1.05
	Interactions	0.99	0.750	0.96	1.03
Diagnosis of mental and behavioral disorders (ICD-10: F04-F69) (yes)	Inappropriate drug choice	1.66	**0.001**	1.24	2.21
	Administration and compliance	1.08	0.677	0.75	1.56
	Dosage	1.59	**0.005**	1.15	2.21
	Adverse drug events	1.30	0.332	0.76	2.22
	Interactions	1.25	0.113	0.95	1.63

Multivariate logistic regression analysis (446 participants assigned to 90 clusters) with GP as random effect variable. For each main group of DRP, there exists LR-*χ*^2^ and *p*: Problems with inappropriate drug choice: *χ*^2^(10) = 33.30, *p* < 0.001; Problems with administration and compliance: *χ*^2^(10) = 16.56, *p* < 0.085; Problems with dosage: *χ*^2^(10) = 17.39, *p* < 0.066; Problems with adverse drug events: *χ*^2^(10) = 19.38, *p* = 0.036; Problems with interactions: *χ*^2^(10) = 56.15, *p* < 0.001. Data presented as mean ±standard deviation or *n* (%). OR, odds ratio; CI, confidence interval; MMSE, Mini-Mental State Examination (range 0–30; higher score indicates better cognitive functioning); B-ADL, Bayer Activities of Daily Living Scale (range 0–10; lower score indicates better performance); GDS, Geriatric Depression Scale (sum score 0–15; score ≥6 indicates depression); bold *p*-values indicate *p* < 0.05.

**Table 4.** Factors associated with total number of DRPs

		bootstrap				
	*b*	Std. Err.	*z*	*p*	95% CI	
Age	−0.002	0.01	−0.37	0.709	−0.02	0.01
Sex (female)	0.08	0.07	1.20	0.233	−0.05	0.21
Cognitive impairment (MMSE)	0.01	0.01	1.38	0.170	−0.00	0.02
Functional status (B-ADL)	−0.005	0.02	−0.34	0.738	−0.04	0.03
Depression (GDS) (yes)	0.13	0.09	1.52	0.131	−0.04	0.31
Total no. of drugs	0.07	0.01	6.62	**0.001**	0.05	0.09
Support with medication	−0.09	0.10	−0.97	0.336	−0.29	0.10
Comorbid diagnoses	−0.001	0.01	−0.29	0.772	−0.01	0.01
Diagnosis of mental and behavioral disorders (ICD-10: F04-F69) (yes)	0.09	0.03	3.06	**0.003**	0.03	0.15
Living alone (yes)	−0.03	0.07	−0.50	0.616	−0.17	0.10
Diagnosis of dementia (ICD-10: F00-F03/G30/G31) (yes)	0.02	0.07	0.31	0.757	−0.11	0.15

Multivariate Poisson regression analysis (446 participants assigned to 90 clusters) with GP as random effect variable: *F*(11.89) = 6.72, *p* < 0.001. Confidence intervals were estimated via the jackknife procedure. CI, confidence interval; MMSE, Mini-Mental State Examination; B-ADL, Bayer Activities of Daily Living Scale; GDS, Geriatric Depression Scale; bold *p*-values indicate *p* < 0.05; *z*, *z*-statistic (derived by dividing the regression coefficient by its standard error).

In the multivariate Poisson regression analysis, the total number of drugs taken (*b* = 0.07; 95% CI: 0.05–0.09; *p* < 0.001) and the presence of a diagnosis of mental and behavioral disorders (*b* = 0.09; 95% CI: 0.03–0.15; *p* = 0.003) were associated with total number of DRPs (significant regression model: *F*(11,89) = 6.18, *p* < 0.001; see Table 4). Cognitive impairment was not associated with the total number of DRPs. The multivariate negative binomial regression analysis provided similar results (see Table S3 availabled as supplementary material online attached to the electronic version of this paper at http://journals.cambridge.org/ipg).

## Discussion

We reported the prevalence and correlates of DRPs in a large sample of community-dwelling primary care patients in Germany who screened positive for dementia. In our setting, 93% of the study participants had at least one DRP. Our findings are in line with a Swedish randomized controlled clinical trial assessing patients aged ≥75 years living in nursing homes or the community and receiving municipal healthcare. They reported the same DRP prevalence of 93% for 182 patients (a mean of 2.5 DRPs per patient, SD 1.5) as in our study (Milos *et al.*, 2013). Our results for the polypharmacy subgroup (67% of the DelpHi-MV study participants with five or more prescription medications) are comparable to the 95% prevalence of at least one DRP observed in a recent analysis of participants from senior centers and residential facilities in the USA aged 60 years and older (mean age 75.9 ± 8.5) taking five or more medications (O'Connell *et al.*, 2015). A high number of drugs taken increase the number of DRPs in persons both with dementia and without dementia, as has been shown in previous studies (Lau *et al.*, 2010; Kaufmann *et al.*, 2015; Lavan and Gallagher, 2016). The majority of the study participants (67%) in our analysis were patients with polypharmacy, this prevalence rate is higher compared to the prevalence in geriatric ambulatory care population in Germany (27%) (Junius-Walker *et al.*, 2007) and worldwide (29%–59%) (Fialova *et al.*, 2005; Lau *et al.*, 2010). Our findings fall in the upper range of prevalence rates found in previous studies of community-dwelling older adults with dementia (45%–73%) (Lau *et al.*, 2011; Oesterhus *et al.*, 2016). The results of the multivariate regression analysis confirmed a strong association of the total number of drugs taken with the occurrence of DRPs.

### Problems caused by administration and compliance

The majority of DRPs were related to administration and compliance (59% of all detected DRPs). Some of these DRPs (inadequate storage, multiple drug taking, or no medication list) can only be found by visiting patients’ home. We found that 41% of the study participants stored their medication inadequately. Thus, medication was exposed to moisture or light or was scattered around the house and, hence, poorly traceable. Critical was the inadequate storage of medications in refrigerators, which occurred by 2.5% of the study participants. This included both the medications that did not need the refrigeration and the refrigerated medications stored outside the refrigerator. Importantly, 1.6% of the study participants with insulin-dependent diabetes stored their insulin outside a refrigerator. The inappropriate storage of medication was most common in patients with polypharmacy. Our findings strongly support the notion that home-based medication review is required to amend a high number of administration- and compliance-related drug problems. In our cohort, 18% of the participants reported in the structured interview that they “often” forget to take their medications; 5% of the participants indicated that they took their medication more often than necessary. This is in line with the results by Elliott where 14% of elderly Australian patients admitted to regularly forgetting to take medication (Elliott, 2006). Adherence is difficult to detect objectively even by a home visit and is underreported (DiMatteo, 2004). The recent systematic review of seven European and U.S. studies revealed that the prevalence of non-adherence in elderly patients living at home ranged from 6% to 55%, and was associated with poor cognition and higher number of drugs taken (Zelko *et al.*, 2016). The same review indicated that there are problems with tools used for the assessment of adherence. We found that inappropriate timing of drug applications occurred in 40% of the study's participants. The term “Incorrect timing of application” in our analysis included both the time of day and the relation to food (with breakfast, an empty stomach, etc.). Incorrect timing of applications was identified in those drugs for which the correct time of intake is important for safety and efficacy of the therapy (e.g. alendronate, levothyroxine, acetylsalicylic acid, and statins). In this analysis, 25% of study participants had no medication list or the medication list was outdated according to the assessment of the study nurse. We cannot estimate if this rate of PwD is high or low because in German studies, the rate of elderly patients with polypharmacy who do not have a medication list differs greatly between 10% and 75% (Jäger *et al.*, 2017). Nevertheless, these findings are relevant because the study participants took six prescribed drugs on average. The absence of a medication list could contribute to a high number of problems caused by administration and compliance in our study sample.

### Problems with drug interactions

In our analysis, problems with potential drug interactions were the second most common category (17% of all detected DRPs), which reflects the high number of drugs taken – on average, each participant took 6.4 prescribed drugs chronically. Accordingly, the total number of drugs was significantly associated with drug interactions in the multivariate analysis. Furthermore, support with medication intake from a caregiver or professional care service was significantly associated with more drug interactions. Our interpretation of this finding is that support for medication intake is more frequent in people with diseases that are treated with medications with high interaction potential. More than one-third (35%) of the DelpHi-MV study participants used at least one drug combination that could potentially lead to a drug interaction of moderate severity. This is in line with the results by Oesterhus *et al.* where 36% of community-dwelling people with mild dementia in Norway (Oesterhus *et al.*, 2016) had drug interactions. Another analysis with the elderly general population in six European countries showed a higher proportion of drug interactions: 46% of the patients had at least one potential drug interaction (Björkman *et al.*, 2002). With increasing number of diseases to treat, the likelihood of drug interaction must be weighed against the necessity to treat a given disease. The information about drug interactions should be used for careful monitoring in this vulnerable population, and for the planned reduction of polypharmacy. In this analysis, a potential clinically relevant drug interaction was detected in 3% of the study participants; this rate is slightly higher than the results of Oesterhus *et al.* (less as 2%) (Oesterhus *et al.*, 2016).

We found that 2% of the study participants took cholinesterase inhibitors and anticholinergic drugs at the same time. Antidementia drugs should not be co-administrated with anticholinergic drugs due to the risk of the effect elimination. Physicians should avoid this combination, and PwD and their caregivers should be sensitized for the use of drugs with anticholinergic properties.

### Problems with inappropriate drug choice

The most common problems with inappropriate drug choice were PIMs according to the Priscus list (23% of study participants received at least one PIM) and the double prescription of active agents (7% of study participants). The prescription rate of PIMs was comparable to the rates found in the general elderly population in Germany (20%–29%) (Zimmermann *et al.*, 2013). The most frequently prescribed PIMs were antidepressants, benzodiazepines, and analgesics. The prevalence of double prescriptions in previous studies varies by setting and method. Community pharmacists detected a lower prevalence of double prescriptions in the German general population (approximately 2% of the patients visiting pharmacies) (Nicolas *et al.*, 2013). Based on an Austria's prescription data set a study reported that up to 15% of the patients receive double prescriptions by different prescribers (Heinze *et al.*, 2016) and 25% to 40% percent of community-dwelling older Australians are prescribed at least one PIM (Elliott, 2006). The diagnosis of mental and behavioral disorders was significantly associated with problems of inappropriate drug choice. This can be explained by the fact that many psychopharmaceuticals which are used to treat mental and behavioral disorders are included in the PIM lists.

### Problems with the dosage

In this analysis, for 8% of the patients, the dosage of drugs was too high according to the current recommendations and guidelines. This prevalence might be overestimated, because a high dosage (beyond the recommendations of the guidelines) could be therapeutically justified. In 5% of participants, the medication review provided evidence that the dosage may be too low. This was particularly the case for antidementia drugs, such as cholinesterase inhibitors and memantine. However, final judgment of these numbers is not possible since the medication review had no information on the tolerance of antidementia drugs for the individual patient which may have been clinically justified. The effective doses of antidementia drugs are known (16–24 mg/24 hours of galantamine, 10 mg/24 hours of donepezil, 6–12 mg/24 hours of rivastigmine or 9.5 mg/24 hours of rivastigmine patch, 20 mg/hours of memantine). The assessment of dosage is an important part of a comprehensive medication review to identify the inappropriate dosing.

### Problems with adverse drug events

In our analysis, 6% of the study participants reported ADEs related to a prescribed medication. This finding is in line with a previous study of Gurwitz *et al.*, who reported a 5% frequency of ADEs in a population of outpatients aged 60 years and older (Gurwitz *et al.*, 2003). The proportion of outpatients with at least one ADE ranged from 5% to 35% in previous studies (Gandhi *et al.*, 2003; Roughead *et al.*, 2004; Elliott, 2006), our results were in the lower range. The number of self-reported ADEs in our analysis can be underestimated because patients with moderate and severe cognitive impairment (22% and 2% of participants in our study, respectively) more frequently had difficulties in communicating their ADEs (Maidment *et al.*, 2008). This assumption was supported by the results of our multivariate logistic regression analysis for different groups of DRPs, results show that the study participants who had better cognitive status reported ADEs more frequently. The validity of self-reported ADE's during the home medication assessment is problematic. It is also possible that some ADEs were not recognized or documented by the study nurses. The recent Australian study showed, that 16% of hospitalized population had a confirmed ADE, half the ADEs were detected after the patient had been admitted and the most were detected by the medical practitioners (Phillips *et al.*, 2014). In our analysis, the lack of GP evaluations of ADEs should be taken into account in the interpretation of the results.

### Limitations

The number of DRPs may be underrepresented in our analysis if the pharmacists did not detect all possible DRPs. Another limitation is the study's dependence on self-reported medication administration and ADEs. There was no additional monitoring of drug administration or comparison of self-reported ADEs with the physician records; thus, our results may underestimate the actual numbers. Patients who were excluded from the analyses due to missing data had more severe cognitive impairment than did the patients who were included. Accordingly, the present study included mostly patients with mild dementia and only a few patients with severe dementia. Effects of severe dementia on DRPs might be underrepresented.

## Conclusion

Our results confirm a high prevalence of DRPs in community-dwelling primary care patients who screened positive for dementia. Cognitive impairment was not a risk factor for an increased number of DRPs. However, the presence of a diagnosis of mental and behavioral disorders was associated with an increased total number of DRPs. In line with earlier studies, our study showed, that a high number of drugs taken is associated with an increased number of DRPs (Lau *et al.*, 2010; Maher *et al.*, 2014; Kaufmann *et al.*, 2015; Oesterhus *et al.*, 2016). Home medication assessment by trained nurses provides benefits for patients with dementia and complex medication regimens, because it gives comprehensive information on the actual medications taken, including OTC drugs, nutritional factors, medication storage, having no or an outdated medication list, no intake due to forgetfulness, multiple drug taking, and self-omission of the drug by the patient. It reflects the real home medication situation more than just checking a medication list at the doctor's office or at the pharmacy. Our data suggest that it cannot be taken for granted that a prescribed drug is taken at the right dosage by the right person and at the right time. Consistent with a range of previous studies, we support comprehensive medication review in PwD and complex medication regimens as part of routine care to avoid harm to patients and to reduce the costs incurred by DRPs in healthcare systems worldwide.

## Conflict of interest

None.

## Description of authors’ roles

D. Wucherer supervised the data collection and drafted the paper. D. Wucherer, I. Kilimann, C. A. Ritter, and S. Teipel were responsible for pharmaceutical analysis. T. Eichler assisted with writing the paper, I. Zwingmann, A. Dreier-Wolfgramm, and B. Michalowsky contributed to the paper in their fields of expertise. J. Hertel and S. Richter were responsible for the statistical analyses. J. R. Thyrian is the coordinator of the study and contributed to the overall design. W. Hoffmann is the principal investigator of the study and has contributed substantially to the concept of this study. All authors have read and approved the final version of the paper.

## Supplementary material

For supplementary material accompanying this paper visit http://dx.doi.org/10.1017/S1041610217001442. click here to view supplementary material
