# Supplementary material for: Drug-related problems in community-dwelling primary care patients screened positive for dementia
Source: Int Psychogeriatr. 2017 Aug 7;29(11):1857–68. doi: 10.1017/S1041610217001442 (PMC5647675; doi:10.1017/S1041610217001442)
Supplement: Supplementary file 1 [file S1041610217001442sup001.zip › S1041610217001442sup001.docx]

## (.docx; 16KB)

## *Supplementary Table 1. Comparison of patients assessed at baseline and those who dropped out before baseline assessment.*

|  | Included  n=516 | Drop-outs  n=118 | t | df | p |
| --- | --- | --- | --- | --- | --- |
| Age (years), mean (SD) | 80.0 (5.5) | 80.2 (5.8) | -0.28 | 170.6 | 0.779^a^ |
| Sex (female), n (%) | 307 (82.3) | 66 (17.7) |  |  | 0.534^b^ |
| DemTect score, mean (SD) | 5.8 (2.1) | 5.5 (2.2) | 1.52 | 171.1 | 0.131^a^ |

Standard deviations or percentages are in brackets. DemTect, range in sample 0-8, higher score

indicates better cognitive functioning; ^a^ Welch’s-t-test; ^b^ Fisher’s exact test; df, degrees of freedom; t, t-statistic of the Welch’s t-test.
