# Supplementary material for: Drug-related problems in community-dwelling primary care patients screened positive for dementia
Source: Int Psychogeriatr. 2017 Aug 7;29(11):1857–68. doi: 10.1017/S1041610217001442 (PMC5647675; doi:10.1017/S1041610217001442)
Supplement: Supplementary file 1 [file S1041610217001442sup001.zip › S1041610217001442sup002.docx]

## (.docx; 17KB)

## *Supplementary Table 2. Differences between patients included in the analyses and those excluded due to missing data (not due to drop out).*

|  | Included | Not included due to missing data | t | df | p |
| --- | --- | --- | --- | --- | --- |
| Age (years), mean (SD) | n=446  79.8 (5.43) | n=70  81.0 (6.18) | -1.51 | 87.03 | 0.136 ^a^ |
| Sex (female), n (%) | n=446  257 (83.7) | n=70  50 (16.3) |  |  | 0.036^b^ |
| DemTect score, mean (SD) | n=446  6.1 (1.90) | n=70  4.5 (2.08) | 4.86 | 80.82 | **0.001^a^** |
| DRP, mean (SD) | n=446  2.79 (1.89) | n=43  3.02 (2.08) |  |  | 0.478^a^ |

Standard deviations or percentages are in brackets. DemTect, range in sample 0-8, higher score

indicates better cognitive functioning; ^a^ Welch’s-t-test; ^b^ Fishers’s exact test; bold p-value indicate p<0.05; df, degrees of freedom; t, t-statistic of the Welch’s t-test.
