# Supplementary material for: Drug-related problems in community-dwelling primary care patients screened positive for dementia
Source: Int Psychogeriatr. 2017 Aug 7;29(11):1857–68. doi: 10.1017/S1041610217001442 (PMC5647675; doi:10.1017/S1041610217001442)
Supplement: Supplementary file 1 [file S1041610217001442sup001.zip › S1041610217001442sup003.docx]

## (.docx; 19KB)

*Supplementary Table 3. Factors associated with DRPs.*

|  | b | Bootstrap Std. Err. | z | p | 95% CI | |
| --- | --- | --- | --- | --- | --- | --- |
| Age | -0.003 | 0.01 | -0.52 | 0.603 | -0.01 | 0.01 |
| Sex (female) | 0.07 | 0.06 | 1.11 | 0.268 | -0.05 | 0.20 |
| Cognitive impairment (MMSE) | 0.01 | 0.01 | 1.29 | 0.196 | -0.00 | 0.02 |
| Functional status (B-ADL) | -0.005 | 0.02 | -0.35 | 0.727 | -0.04 | 0.02 |
| Depression (GDS) (yes) | 0.13 | 0.08 | 1.67 | 0.094 | -0.02 | 0.29 |
| Total no. of drugs | 0.07 | 0.01 | 7.25 | **0.001** | 0.05 | 0.09 |
| Support with medication | -0.11 | 0.08 | -1.35 | 0.178 | -0.26 | 0.05 |
| Comorbid diagnoses | -0.002 | 0.01 | -0.30 | 0.763 | -0.01 | 0.01 |
| Diagnosis of mental and behavioral disorders (ICD-10: F04-F69) | 0.09 | 0.04 | 2.55 | **0.011** | 0.02 | 0.16 |
| No. of Persons living in household^d^ | 0.004 | 0.05 | 0.09 | 0.931 | -0.09 | 0.09 |
| Diagnosis of dementia (ICD-10: F00-F03/G30/G31) | 0.02 | 0.06 | 0.36 | 0.722 | -0.10 | 0.15 |

Multivariate negative binomial regression analysis (n=446 patients assigned to n=90 clusters) with GP as random effect variable: Wald chi^2^(11)= 82.95, p<0.001. Confidence intervals were estimated via the jackknife procedure. CI, confidence interval; MMSE, Mini Mental State Examination; B-ADL, Bayer Activities of Daily Living Scale; GDS, Geriatric Depression Scale; ^d^=Model did not converge for the dichotomized living alone variable, thus the metric variable No. of Persons living in Household was included. Bold p-values indicate p<0.05; z, z-statistic (derived by dividing the regression coefficient by its standard error).
